# Natural product nobiletin-loaded Pickering emulsion stabilized by bovine serum albumin/carboxymethyl inulin complexes: preparation and digestive characteristics

**DOI:** 10.3389/fphar.2024.1375779

**Published:** 2024-05-01

**Authors:** Guiying Huang, Man Zhang, Konglong Feng, Jie Xiao, Qingrong Huang, Chi-Tang Ho, Jun Liu

**Affiliations:** ^1^ College of Light Industry and Food Science, Zhongkai University of Agriculture and Engineering, Guangzhou, Guangdong, China; ^2^ Department of Food Science, Rutgers University, New Brunswick, NJ, United States; ^3^ College of Food Science, South China Agricultural University, Guangzhou, Guangdong, China; ^4^ College of Food Science and Engineering, Yangzhou University, Yangzhou, Jiangsu, China

**Keywords:** complexes, Pickering emulsion, nobiletin, 4-demethylnobiletin, bioavailability

## Abstract

To expand the application of nobiletin (NOB) in semi-solid functional foods, bovine serum albumin (BSA)/carboxymethyl inulin (CMI) complexes-stabilized Pickering emulsion (BCPE) (φ_oil_ = 60%, v/v) was fabricated, and the swallowing index and bioavailability of the NOB-loaded Pickering emulsion was evaluated. Confocal laser scanning microscope (CLSM) and cryo-scanning electron microscopy (cryo-SEM) images revealed that BSA/CMI complexes attached to the oil–water interface. NOB-loaded BCPE exhibited a viscoelastic and shear-thinning behavior. Fork drip test results suggested that the textural value of unloaded and NOB-loaded emulsions was International Dysphagia Diet Standardisation Initiative Level 4, which could be swallowed directly without chewing. The *in vitro* lipolysis model suggested that NOB had a faster digestive profile and a higher bioaccessibility in the BCPE than in the oil suspension. The *in vivo* rat model revealed that the oral bioavailability of NOB was increased by 2.07 folds in BCPE compared to its bioavailability in unformulated oil. Moreover, BCPE led to a higher plasma concentration of the major demethylated metabolite of NOB (4′-demethylnobiletin) than the unformulated oil. Accordingly, BCPE enhanced the oral bioavailability of NOB by improving bioaccessibility, absorption, and biotransformation.

## Graphical Abstract

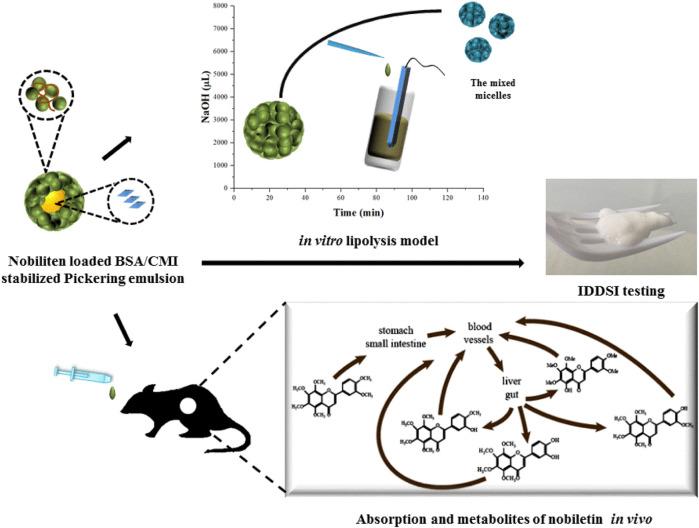

## Highlights

- A bovine serum albumin/carboxymethyl inulin complexes-stabilized Pickering emulsion (BCPE) with a 60% oil content was fabricated. The textural values of unloaded and nobiletin (NOB)-loaded BCPE were International Dysphagia Diet Standardisation Initiative (IDDSI) Level 4.
- BCPE enhanced the oral bioavailability of NOB in rats compared to oil suspension.
- BCPE improved the biotransformation of 4′-demethylnobiletin *in vivo* during 24 h.

## 1 Introduction

Natural products are important sources of clinical drugs and functional food owing to their bioactivities and low toxicity (Li et al., 2022; Zhang et al., 2022; Wu et al., 2023). Nobiletin (NOB; 5,6,7,8,3′,4′-hexamethoxyflavone) is a major component of citrus polymethoxyflavones (PMFs), which are a group of flavonoids predominately found in citrus peels (Li et al., 2006a; Zhang et al., 2021). NOB has healthcare functions due to its profound bioactivities, such as anticancer, anti-inflammation, anti-atherosclerosis, anti-viral, and neuroprotective effects, and its ability to prevent neurodegenerative diseases and regulate lipid metabolism, glucose metabolism, mitochondrial function, and muscle physiology (Gao et al., 2018; Nakajima and Ohizumi, 2019; Nohara et al., 2019). These bioactivities can be achieved by the blood circulation system *in vitro* (Chen et al., 2020; Min et al., 2024). *In vivo* experiments infer that NOB and its metabolites are distributed to different tissues via the blood circulation system and ultimately excreted in urine. NOB undergoes phase I and II reactions in the small intestine; the major metabolites of NOB in the urine of mice are demethylated products of B-ring methoxyl groups, including 4′-demethylnobiletin (4′-DMN), 3′-demethylnobiletin (3′-DMN), and 3′,4′-didemethylnobiletin (3′,4′-DDMN; Zhang et al., 2020). Among these metabolites, 4′-DMN is the major metabolite of NOB and has a higher anti-inflammatory and immunomodulatory therapeutic effect than the parent NOB (Li et al., 2006b; Li et al., 2014; Wu et al., 2015; Zheng et al., 2015; Huang et al., 2016; Wu et al., 2018). Therefore, NOB is suitable for the functional enhancement of food. However, NOB exhibits a bitter taste that hampers its product acceptance and limits its application in the functional food area (Batenburg et al., 2016). Specific food formulations must be developed to expand the application of NOB in semi-solid functional foods. Although formulation can reduce the bitterness of NOB, it changes the oral bioavailability of NOB. Evaluation of the oral and digestive characteristics of any novel formulations of NOB should be performed.

A complexes-stabilized Pickering emulsion is one type of emulsion stabilized by particles. The complexes are formed by electrostatic interactions, which are physical reactions that do not change the structure of raw materials. A complexes-stabilized Pickering emulsion has various advantages, such as high loading, high biocompatibility, and desired viscoelasticity, and it is being composed of functional biopolymers and small molecules (Dickinson, 1998; Jie et al., 2016). Owing to nutrient density, remodeling ability, and easy-to-swallow. Complexes-stabilized Pickering emulsion may be a novel food matrix for special populations (Goldstein et al., 2017; Kerien et al., 2020). A complexes-stabilized Pickering emulsion is made with a simple process, and its textural properties can be shifted by adjusting the raw materials (Ming et al., 2023). 

Complexes-stabilized Pickering emulsions can be fabricated in two steps. First, complexes are prepared via electrostatic interaction between two types of biomacromolecules with opposite charges. Then, the complexes stabilize the Pickering emulsion by providing steric and electrostatic repulsion or reducing interface tension. Although many studies focus on the development of complexes-stabilized Pickering emulsions, the texture and digestive characteristics of the resulting emulsions have rarely been reported. Emulsion systems used to obscure the bitterness of natural products have been reported. Some emulsions can distract off-flavor or trigeminal effects by their ingredients, while others can reduce the contact time between natural products and taste buds by being swallowed directly without chewing (Batenburg et al., 2016). To define the swallowing characteristics of foods, the International Dysphagia Diet Standardisation Initiative (IDDSI) developed the IDDSI framework. This framework can be applied to evaluate the swallowing characteristics of emulsions.

Inulin is a fructan-type plant polysaccharide derived from inulin or chicory. Inulin has many biological activities, such as antioxidant and anticancer effects; regulation of blood sugar, blood lipids, and the immune system; and improvement of intestinal health, and benefits for metabolic syndromes (Wan et al., 2020). Moreover, because inulin has low non-specific absorption in most tissues and can be easily filtered by kidneys, it has been used in the pharmaceutical and food industries for decades. Carboxymethyl inulin (CMI) is a derivative of inulin that changes surface electric charge, increases solubility, and decreases viscosity (Joshi et al., 2017).

Our previous work revealed that CMI and bovine serum albumin (BSA) could form complexes induced by physical interactions (Huang et al., 2019). In this study, we used BSA/CMI complexes to stabilize a Pickering emulsion. The resulting BSA/CMI complexes-stabilized Pickering emulsion (BCPE) was applied to encapsulate NOB. The bioaccessibility of NOB-loaded in BCPE was measured using an *in vitro* lipolysis model. The oral bioavailability of NOB was monitored using a rat model to compare the formulated form and an unformulated oil suspension. In addition to evaluating the oral bioavailability of NOB *in vitro* and *in vivo*, the biotransformation of NOB in rats is discussed.

## 2 Materials and methods

### 2.1 Materials

BSA (>98%) was purchased from Sigma-Aldrich Chemical Co. (St. Louis, MO, USA). CMI was synthesized in our laboratory (Rutgers University, NJ, USA) (Huang et al., 2019). Medium-chain triglyceride (MCT) was requested from Stepan Company (Northfield, IL, USA). NOB and tangeretin (98% purity) were obtained from Shanxi Huike Plant Development Co., Ltd. (Shanxi, China). 3′,4′-DDMN, 3′-DMN, and 4′-DMN were synthesized and identified using LC-QTOF-MS/MS (Zhang et al., 2020). Hydrochloric acid (HCl) was purchased from Fisher Scientific (Waltham, MA, USA) (Zhang et al., 2020). Tris was purchased from Saiguo Biotechnology Co., Ltd. (Guangdong, China). Maleic acid was purchased from America SECOMA Biotechnology Co., Ltd. (Beijing, China). Calcium chloride was purchased from Damao Chemical Co., Ltd. (Tianjin, China). Sodium taurodeoxycholate (NaTDC) was purchased from Ruiyong Biotechnology Co., Ltd. (Shanghai, China). Lecithin from soybean, pancreatin, and Tween 80 were purchased from Macklin Biochemical Co., Ltd. (Shanghai, China). HPLC-grade acetonitrile (ACN) and methanol were purchased from Gris Pharmaceutical Chemical Technology Co., Ltd. (Tianjin, China). HPLC-grade tetrahydrofuran, ammonium acetate, trifluoroacetic acid (TFA), and acetic acid were purchased from Yien Chemical Technology Co., Ltd. (Shanghai, China). Dimethyl sulfoxide (DMSO), β-D-glucuronidase, and sulfatase were purchased from Sigma-Aldrich Chemical Co. (St. Louis, MO, USA). Milli-Q distilled water was used in all experiments.

### 2.2 Formation of BSA/CMI complexes

BSA (1% w/v) and CMI (0.2% w/v) were mixed in water solutions free from NaCl. After being fully dissolved for 1 h using a magnetic stirrer, the solution was adjusted to pH 4.0 using HCl solution, and a suspension of BSA/CMI complexes was obtained. The mean particle size of the BSA/CMI complexes was determined using a Brookhaven 90 Plus/BI-MAS instrument equipped with a 15-mW solid-state laser for multi-angle particle sizing. The BSA/CMI complexes were diluted 10 times using water (pH 4.0) and dispersed for 30 min using an ultrasonic oscillator (40 kHz) before particle size measurement.

### 2.3 Fabrication of unloaded and NOB-loaded BSA/CMI complexes-stabilized Pickering emulsion

The aqueous phase was prepared as follows. BSA (10 mg/mL) and CMI (2 mg/mL) were fully dissolved in 5 mL distilled water, and the solution was adjusted to pH 4.0. The aqueous phase was homogenized for 2 min at 10,000 rpm using a T25 digital ULTRA-TURRAX^®^ homogenizer (IKA-Werke GmbH & Co. KG, Germany) equipped with an S25N-10G dispersing rotor. The oil phase was slowly added to the aqueous phase. Then, the BCPE with an oil fraction of 60% was obtained after homogenization for 3 min at 10,000 rpm.

The oil phase of NOB-loaded BCPE was prepared as follows. NOB powder of 2% (w/v) was added into 10 mL MCT and stirred at 90°C using an oil bath until the NOB was completely dissolved. Then the MCT solution was cooled to 35°C before being added to the water phase. The concentrations of NOB, BSA, CMI, and MCT were set at 1.2%, 1%, 0.2%, and 60% (w/v), respectively.

### 2.4 Microstructure measurement of BCPE

The microstructures of the BCPE samples were observed on a Zeiss FV120 confocal laser scanning microscope (CLSM) with an inverted microscope (model Leica DM IRB). The oil and the aqueous phases were stained with curcumin (green) and rhodamine B (red), respectively. The CLSM was performed using two laser excitation sources (488 and 543 nm).

Freeze fracture scanning electron microscopy (cryo-SEM) was used to examine the interfacial structure of BCPE droplets. A small sample volume was placed onto a Cu sample stub and snap-frozen in a liquid nitrogen bath. The frozen sample was transferred to a preparation chamber (PP3010T Cryo-SEM preparation system, USA). After being fractured with a cooled knife and subjected to sublimation at 95°C for 9 min, the sample was sputtered and coated with platinum. SEM measurement of the sample was performed using a cold-field emission scanning electron microscope (S-4800 Hitachi, Japan).

### 2.5 Microstructure analysis of NOB-loaded BCPE

The microstructure of NOB-loaded BCPE was analyzed using an inverted microscope with a refrigeration charge-coupled device (CCD) camera (OPTEC DV330). Images (40×) were captured after the sample was placed on a glass microscopic slide.

### 2.6 Rheological properties of NOB-loaded BCPE

Rheological measurement of complexes and *Pickering* emulsion was performed following the method described in Zou (2018). The shear viscosities were determined at 25°C using a TA ARES-G2 rheometer (TA Instruments, New Castle, DE, USA) with parallel plates (d = 20 mm). The shear rate range was set to 1–100 s^−1^. Frequency sweeps were performed from 0.08 to 10 Hz at the strain of the identified linear viscoelastic region. Dynamic strain scanning was performed in the range of 0.01%–100%. All the samples were loaded onto the parallel plate with the gap at 1 mm for 10 min before analysis.

### 2.7 Fork drip test of unloaded or NOB-loaded BCPE

A fork drip test was performed using the IDDSI testing methods (Hadde and Chen, 2020). The IDDSI framework consists of a continuum of eight levels (0–7), where flow tests are used in levels 0–3, while fork drip tests are applied in levels 4–7. Ten participants were provided with pictures and descriptions of IDDSI Levels 3–5. The participants then assigned each sample to an IDDSI category. Each judgment was determined individually without discussion.

### 2.8 Lipolysis analysis of NOB in BCPE and MCT suspension

An *in vitro* lipolysis study was performed using the Ting et al. (2015) method. The lipolysis buffer consisted of tris maleate (50 mM), sodium chloride (150 mM), calcium chloride (5 mM), NaTDC (5 mM), and phosphatidylcholine (5 mM). Pancreatin powder (1 g) was added into 5 mL of lipolysis buffer, and then the well-mixed solution was centrifuged at 2000 rpm for 10 min. The obtained supernatant was stored on ice until further use. The simulated small intestinal fluid (SSIF) was mixed with 1 mL of pancreatin suspension and 9 mL of lipolysis buffer.

For the *in vitro* lipolysis study, a sample containing 250 mg of the oil phase was injected into 10 mL of the SSIF. The mixture was maintained at 37°C ± 1°C and stirred in an oil bath. The pH of the mixture was adjusted to 7.50 ± 0.02 by titrating with 0.25 N sodium hydroxide solution. The temperature and pH were kept constant for 2 h. The consumption of sodium hydroxide solution was recorded at each time point.

After the 2-h lipolysis, the final mixture was ultracentrifuged at 4°C for 60 min at 50,000 rpm using an Optima XE-100 ultracentrifuge (Beckman Coulter Life Sciences, Indianapolis, IN, USA). After ultracentrifugation, the mixture was separated into three layers. The upper layer was the undigested oil phase, the middle transparent layer was the micelle phase containing soluble NOB, and the bottom was the solid precipitant. The micelle phase was tested for NOB concentration by HLPC, and its volume was measured.

A 100-μL aliquot of the micelle phase sample was filtered and added to 400 μL of methanol. The content of NOB in the micelle phase was analyzed using HPLC. The bioaccessibility (%) of NOB was calculated using the following equation:

$$\text{Bioaccessibility }\left(\%\right)=\frac{\text{total}\;\text{mass}\;\text{of}\;\text{solubilized}\;\text{NOB}\;\left(g\right)}{\text{total}\;\text{mass}\;\text{of}\;\text{NOB}\;\text{in}\;\text{original}\;\text{lipid}\;\text{samples}\;\left(g\right)}\times 100\%$$

### 2.9 Animal experiment

The animal experiment was performed according to the previously reported method of Zhang et al. (2020). Young (6 weeks old) healthy male Sprague-Dawley rats were purchased from Southern Medical University (Guangdong, China). During a week of acclimation, all rats were housed in a controlled environment (about 25°C and 40%–60% relative humidity) with a 12-h light–dark cycle. All rats were fed with Purina Laboratory Chow 5001 and *ad libitum* water. Care of the rats followed the Chinese Government’s Guide for the Care and Use of Laboratory Animals. The experimental protocol (protocol number: 2019048) was approved by the Institutional Animal Care and Use Committee of South China Agricultural University.

Before conducting a pharmacokinetics study, the rats (around 250 g) were fasted overnight and randomly divided into four groups (five rats in each group). Rats in the first group were orally administered 100 mg/kg NOB-loaded BCPE using oral gavage, while rats in the second group were orally administered 100 mg/kg NOB in MCT. Blood samples from eyeballs were collected at 0.5, 1, 2, 4, 6, 10, and 24 h. The whole-blood samples were immediately centrifuged at 5,000 rpm at 4°C for 15 min, and the plasma samples were collected and stored at −80°C before HPLC analysis.

Prior to HPLC analysis, a plasma sample of 200 μL was mixed with 20 μL tangeretin (10 μg/mL, methanol solution) as an internal standard and 20 μL enzymes of β-D-glucuronidase (500 U) and sulfatase (10 U). The mixture was incubated at 37.8°C for 45 min. Then, ethyl acetate of 400 μL was added, and the suspension was vortexed at 3,000 rpm for 3 min. After centrifugation at 10,000 rpm for 5 min, the supernatant was transferred to a centrifuge tube. After repeating the steps, the combined supernatant was dried using flowing nitrogen. Dried samples were dissolved in 150 μL of 80% methanol in water containing 0.2% acetic acid. The solution was filtered with a 0.22-μm filter membrane before HPLC analysis.

### 2.10 UPCL–MS/MS analysis of NOB in plasma of rat

NOB and its metabolites in the rat plasma were identified using UPLC−MS/MS (Shimadzu, Kyoto, Japan) consisting of an LC-30AD and a triple quadrupole linear ion trap mass spectrometer (model QTRAP 4500) (AB SCIEX, Concord, Canada) based on multiple reaction monitoring (MRM). An Agilent Poroshell 120 PFP (4.6 × 150 mm, 2.7 μm) column was used in the detection. The measurement was performed according to the method previously reported by Zhang et al. (2020).

### 2.11 HPLC analysis of NOB in rat plasma

The measurement of NOB concentration in plasma samples was conducted using an LC-20A HPLC system equipped with a PDA (UV-VIS) absorption detector (Shimadzu Corporation, Kyoto, Japan) and an Ascentis RP-Amide reversed-phase HPLC column (15 cm × 4.6 mm id, 3 μm; Sigma-Aldrich, St. Louis, MO, USA), according to the previously reported method of Zheng et al. (2015) with minor modifications. Detection wavelength, column temperature, flow rate, and injection volume were set to 320 nm, 20°C, 1 mL/min, and 30 μm, respectively. The detection of NOB was performed using a gradient elution of two complex mobile phases, A and B. A was 75% water, 20% ACN, 5% THF, and 50 mM ammonium acetate; B was 50% water, 40% ACN, 10% THF, and 50 mM ammonium acetate. The pH values of both mobile phases were adjusted to 3.0 using TFA. The gradient elution using phases A and B began with 10% B, and then increased to 20%, 40%, 60%, 70%, and 100% B at 3, 8, 23, 29, and 32 min, respectively. Then, the volume percent of phase B was reduced to 10% in 3 min, and detection was stopped after 1 min. The total elution time was 36 min.

### 2.12 Statistical analysis

Experiments were conducted in duplicate, and results were expressed as the mean ± standard deviation (SD). Error bars on figures represent standard deviations. The Q test with 90% confidence interval was applied in the rat experiments.

## 3 Results and discussion

### 3.1 Fabrication of BCPE

**FIGURE 1:**
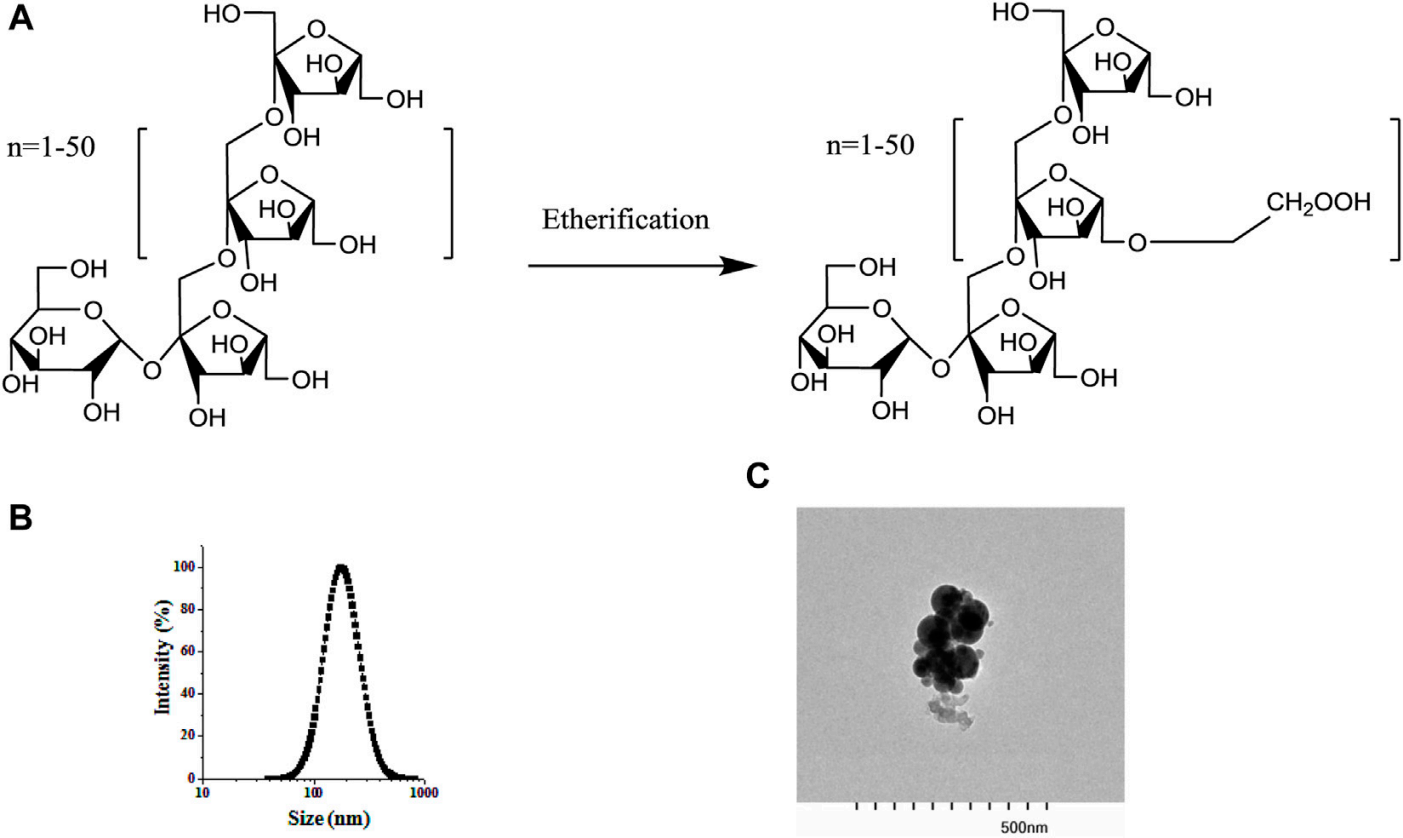
Structure of inulin and CMI **(A)**, particle size distribution of BSA/CMI complexes **(B)**, and TEM images of BSA/CMI complexes (bar scale = 500 nm) **(C)**.

BSA/CMI complexes were prepared before the BCPE was fabricated. CMI samples with 0.5 degrees of substitution were synthesized by etherification technology (Figure 1A). A mixed solution of BSA and CMI complexes at a 5:1 ratio was prepared at pH 4. DLS analysis showed that the average size of the complexes was 200.33 nm. The transmission electron microscope photograph of the complex indicated that the complexes were formed by the aggregation of spherical particles. In our previous study, isothermal titration microcalorimetry (ITC) data revealed that BSA/CMI complexes were dominated by enthalpy changes (∆H < 0, ∆S < 0). Therefore, electrostatic and hydrogen bonding interactions occurred between BSA and CMI. CMI was the bridge for the connection of BSA particles. Raman spectra suggested that the formation of complexes led to the change in the secondary structure of BSA and to the aggregation of BSA (Huang et al., 2019). The BSA/CMI complexes were used to stabilize the Pickering emulsion with an oil phase of 60% (Li and Huang, 2015). Therefore, BCPE was fabricated based on the combined mechanisms of BSA and CMI to prevent flocculation and coalescence (Bouyer et al., 2012).

### 3.2 Microstructures of BCPE

**FIGURE 2:**
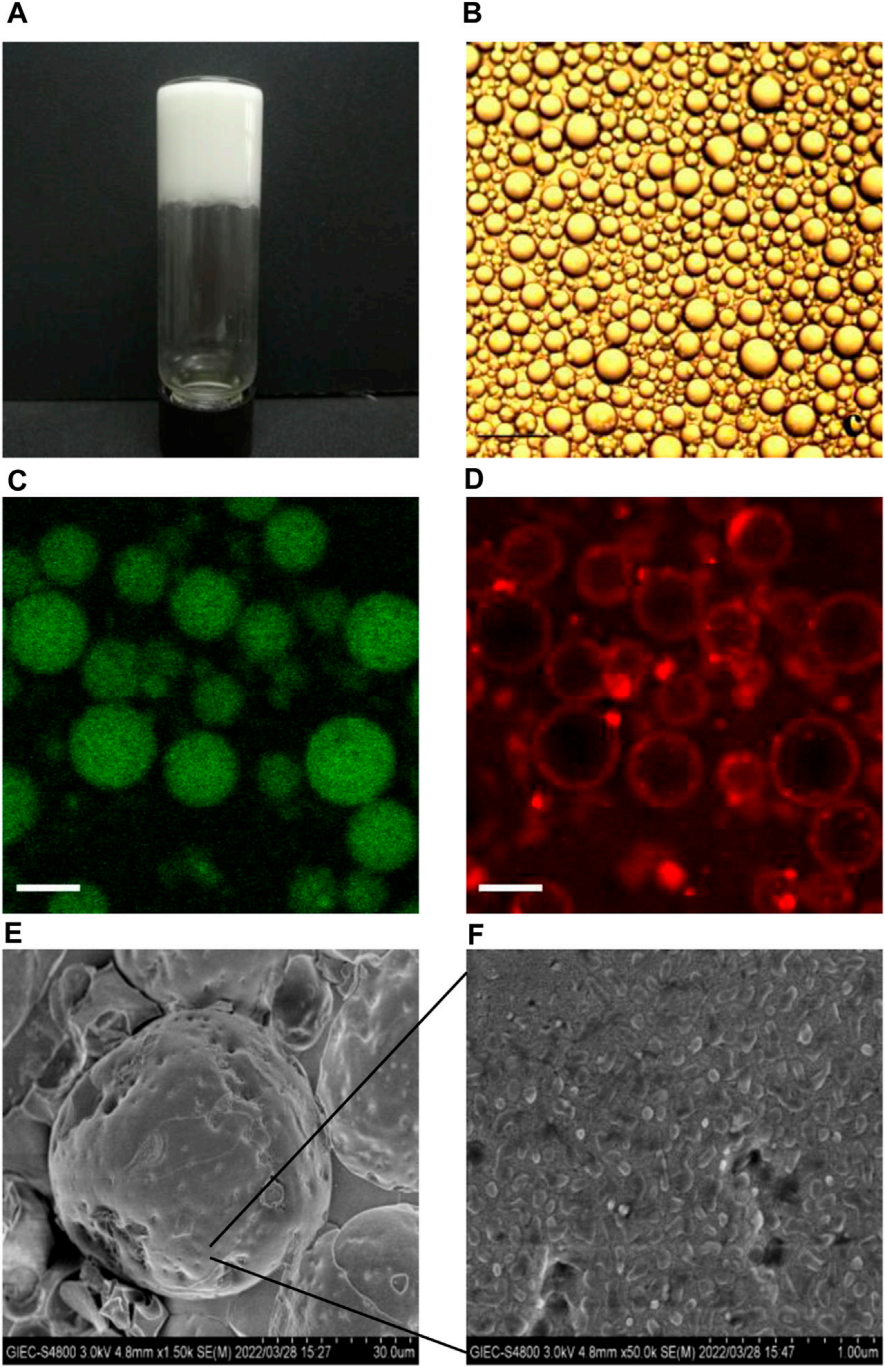
Visual photograph **(A)** and microscopic image **(B)** of NOB BSA/CMI-PE (bar scale = 25 μm); CLSM images of BSA/CMI-PE: excitation at 488 nm **(C)** and excitation at 543 nm **(D).** The oil and water phases were stained with curcumin (green) and rhodamine B (red), respectively (bar scale = 30 μm); cryo-SEM images of BSA/CMI-PE particles **(E)** and the surface of BSA/CMI-PE particles **(F)**.

Figure 2A shows the photographs of BCPEs after 7 days of preservation. A uniform milky gel-like material was observed, suggesting that a stable emulsion was fabricated. The microscope image shows BCPE droplets with an average droplet size of 28 μm. CLSM measurement of BCPE was performed. The oil and aqueous phases were stained with curcumin (green) and rhodamine B (red), respectively. The green globes in Figure 2C indicated that the Pickering emulsion is an O/W emulsion Figure 2D showed that a certain thickness aggregated around the oil droplets. This evidence suggests that the BSA/CMI complexes adsorbed on the surface of oil droplets, and the complexes provided the steric hindrance to stabilize BCPE (Jiang et al., 2021).

The emulsion samples were quickly frozen using liquid nitrogen. The water in the samples cooled rapidly and formed amorphous ice. The emulsion samples were embedded in amorphous ice, and their state was captured in cryo-SEM images. Typical emulsion droplets stabilized by BSA/CMI complexes with indentations are exhibited in Figure 2E. When observed under higher magnification, rod-like nanoscale particles were absorbed onto the surface of emulsion droplets (Figure 2F; Xiao et al., 2016). These particles were larger than pure BSA particles, suggesting that the nanoparticles on the surface of oil droplets were BSA/CMI complexes. Based on the high oil content and droplet density, the distance between the oil droplets is relatively close. The BSA/CMI complexes stabilized the Pickering emulsion by reducing interfacial energy and shielding oil droplets from coalescence.

### 3.3 Textural properties of unloaded and NOB-loaded BCPEs

**FIGURE 3:**
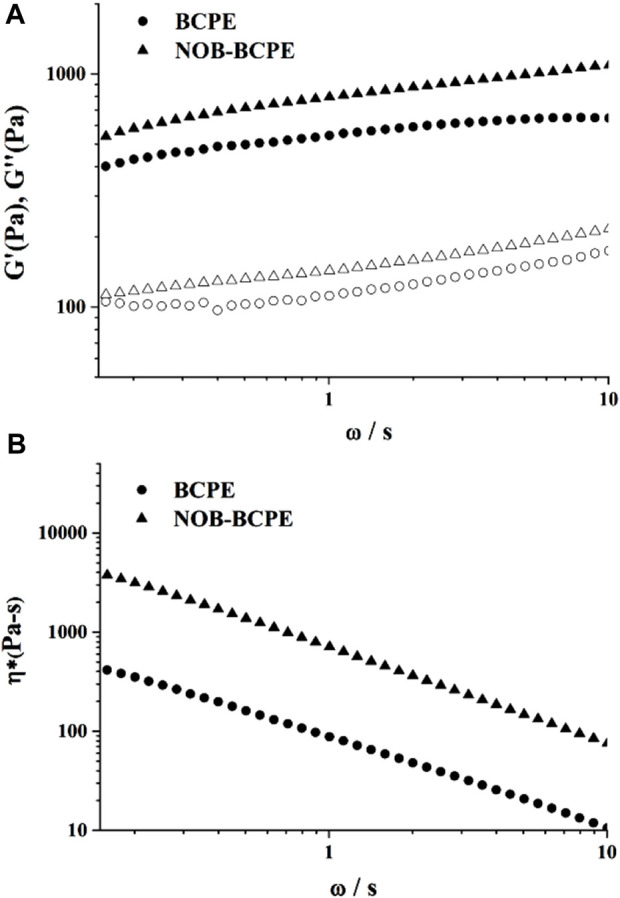
Plots of storage modulus (G′) and loss modulus (G″) *versus* angular frequency for NOB-loaded BSA/CMI-PE **(A)**. Plots of tan δ *versus* angular frequency for NOB-loaded BSA/CMI-PE **(B)**.

An optimal BCPE formulation was used to encapsulate 1% (w/v) NOB. Its stability was evaluated using rheological measurements. Dynamic (oscillatory) measurements of BCPE and NOB-loaded BCPE (NOB-BCPE) were carried out as a function of frequency. Both the storage modulus G′ and the loss modulus G″ of BCPE and NOB-BCPE slightly increased with increased frequency, revealing little dependence on frequency (Figures 3A,B). The significantly higher G′ than G″ at all frequencies indicated that the BCPE and the NOB-BCPE formed an interconnected gel-like network microstructure. The viscoelastic properties of this microstructure led to the uniform distribution of NOB in BCPE (Sun et al., 2018). The complex viscosity of BCPE decreased nearly linearly with frequency, suggesting shear-thinning behaviors. A similar phenomenon was observed in the rheological properties of a NOB-loaded nanoemulsion (Zhang et al., 2020). However, this result was in contrast to the result found with a tangeretin- or a 5-demethyltangeretin-loaded nanoemulsion or a PMF (tangeretin of 66% and NOB of 23%)-loaded whey protein isolate/pectin complexes-stabilized Pickering emulsion (Ting et al., 2013; Wijaya et al., 2021). Therefore, the rheological properties of PMFs-loaded emulsion were probably associated with the type and concentration of PMFs and the different kinds of emulsion systems.

The 10 participants in the fork drip test experiments identified the texture values of unloaded and NOB-loaded BCPEs as IDDSI Level 4. The flowability of the unloaded BCPE was better than that of the NOB-loaded BCPE. The emulsions could be brought to the back of the mouth by the tongue for swallowing without being bitten or chewed. The oral chewing properties of the emulsions could be ignored.

### 3.4 *In vitro* lipolysis model analysis of NOB in BCPE and MCT suspensions

The bioaccessibility of NOB loaded in BCPE and MCT suspensions was evaluated using an *in vitro* lipolysis model study. The absorption of hydrophobic bioactives was positively related to their water solubility and stability in the GI tract (Ting et al., 2015). Lipid-based formulations are developed to improve the water dispersity and enhance the absorption of hydrophobic compounds. After digestion in the GI tract, the lipids would be hydrolyzed and then released as free fatty acids (FFAs). The release of FFAs promoted the formation of mixed micelles. The mixed micelles generated in the small intestine, which consist of FFAs and bile salts, could solubilize hydrophobic compounds for intestinal transport. The solubilization capacity for NOB was enhanced by the increase of mixed micelles.

**FIGURE 4:**
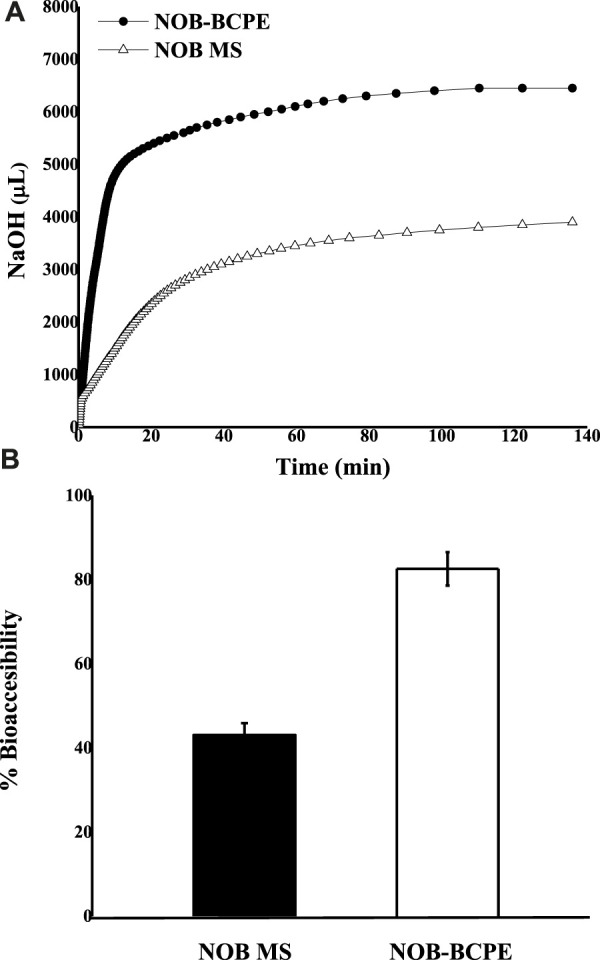
*In vitro* comparison of the lipid digestion kinetics of NOB BCPE and MS expressed as the amount of NaOH added as a function of time **(A)**. Comparison of NOB percent bioaccessibility relative to the original dose in BCPE and MS **(B)**. Data are presented as mean ± standard deviation (n = 3).

In this *in vitro* model, the existing FFAs led to decreased pH in the SSIF. The addition of NaOH could neutralize the FFAs and maintain the pH at an initial value of 7.50 ± 0.02. The consumption of NaOH as a function of time can be used to indicate the rate of lipid digestion (McClements and Li, 2010). Figure 4A shows the consumption of NaOH solution as a function of time; most of the lipid digestion of NOB-loaded BCPE occurred within the first 10 min. By contrast, the BCPE consumed more than three times the amount of NaOH within the first 10 min. After 2 h, the final consumption of NaOH in the BCPE was nearly twice that in the MCT suspension. Figure 4B shows that the bioaccessibility of NOB was much higher in the BCPE (82.55% ± 3.97%) than it was in the MCT (43.04% ± 2.84%). The higher bioaccessibility of NOB in the BCPE resulted from the higher surface area-to-volume ratio and, thus, the greater extent of lipid hydrolysis of the BCPE than of the MCT suspension.

Three major factors caused the difference in FFA release from the BCPE and MCT suspensions. First, BCPE increased the contact area between the oil droplets and the SSIF. Second, the interfacial properties were altered by the BSA/CMI complexes. The complexes absorbed on the droplet surfaces and formed a thicker layer to prevent the flocculation and coalescence of oil droplets in the small intestine digestion fluid (McClements, 2018; Wijaya et al., 2021). Third, although the digested BSA probably released peptides, amino acids, and some protons (H^+^), which might lead to an over-estimate of the actual value of released FFA, the peptides or amino acids could favor the formation of mixed micelles (Wijaya et al., 2020). Accordingly, BCPE could enhance the bioaccessibility of NOB proportionally to the rate and extent of lipid digestion (Ting et al., 2015). Due to the liposolubility at the concentration of 1.2% (w/v), the first-pass effect of NOB could be enhanced.

### 3.5 *In vivo* animal model analysis of NOB in BCPE and MCT suspensions

The biological activity of NOB is associated with intake and bioavailability, depending on its absorption, metabolism, and excretion in the human body. After absorption in the small intestine, NOB underwent phase I and II metabolisms. In these two phases, NOB was converted into sulfates, glucuronides, and methylated metabolites in the small intestine. The metabolites of NOB, consisting of 3′,4′-DDMN, 3′-DMN, and 4′-DMN, formed. The metabolites pass to the portal vein and liver before entering the blood and being excreted in urine (Arshad et al., 2023). Because the Pickering emulsion might lead to a slow release and because its ingredients could affect enzymatic activity, NOB and its major metabolites were identified and detected in serum (Ting et al., 2015; Wan et al., 2020). After a single oral administration of NOB (100 mg/kg) in either BCPE or MCT suspension, the distinctive pharmacokinetic profiles of NOB and its metabolites of 4′-DMN, 3′-DMN, 3′,4′-DDMN, and 5-demethylnobiletin in rats were confirmed by UPLC-MS/MS at 6 h. 3′-DMN and 4′-DMN were further identified by HPLC-DAD. NOB and its metabolites were detected by HPLC-DAD at 0.5, 1, 2, 4, 6, 10, and 24 h.

**FIGURE 5:**
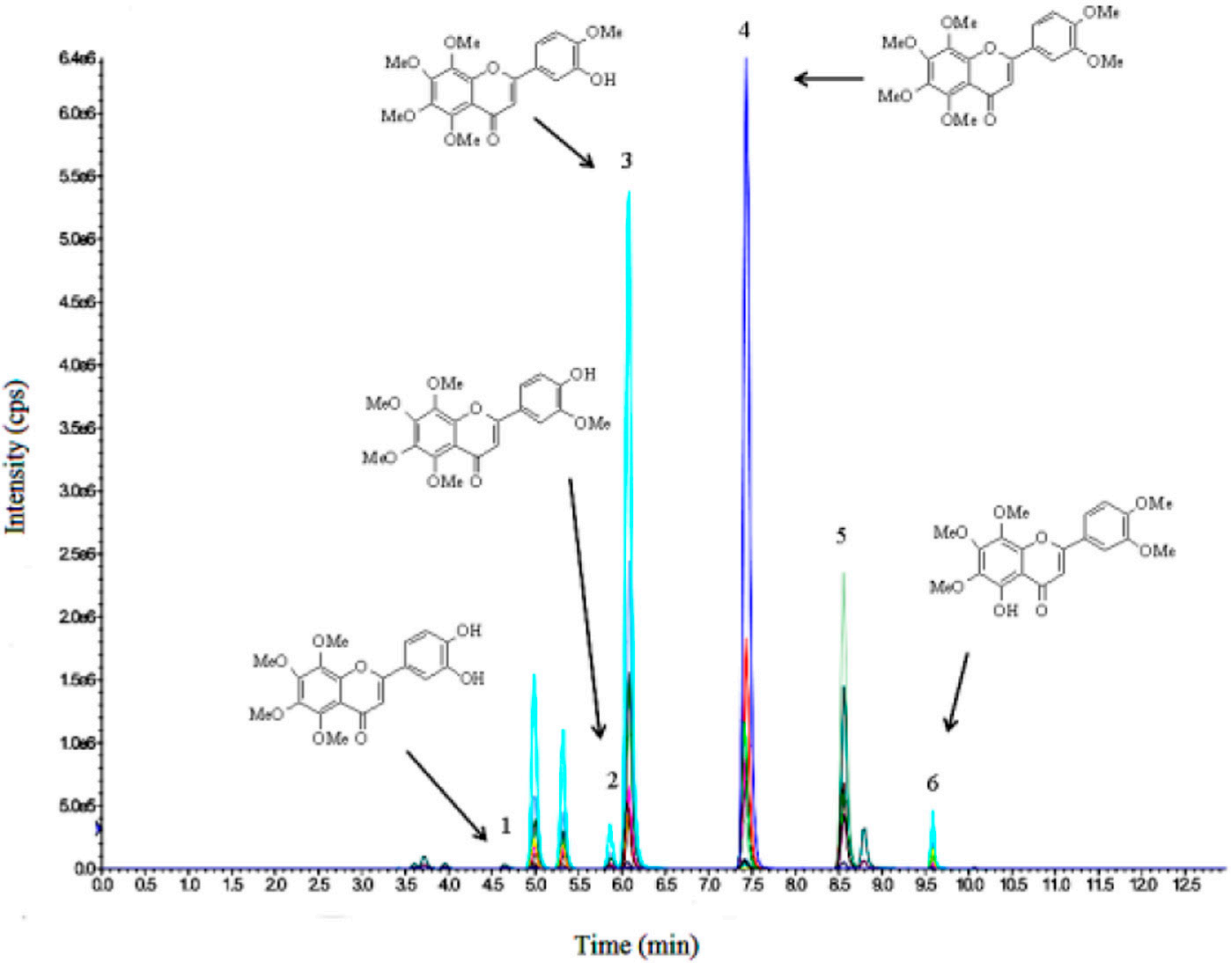
LC-MS/MS profile of 3′,4′-DDMN (1), 3′-DMN (2), 4′-DMN (3), NOB (4), tangeretin (5), and 5-demethylnobiletin (6) in plasma at 6 h after oral administration.

After the conjugates of NOB and its metabolites were treated by enzymes, mass spectral data were obtained with the ESI positive-ion mode under different collision voltages. NOB and its metabolites were observed at 0.5 h after oral administration, suggesting the absorption and metabolism of NOB. NOB and 4′-DMN had the highest concentrations within 24 h in all plasma samples, compared to the other metabolites, illustrating that 4′-DMN was the major metabolite. This result was similar to the metabolites identified in the urine of mice fed with NOB after absorption in the small intestine and liver metabolism (Li et al., 2006b; Zheng et al., 2013). 4′-DMN, 3′-DMN, and 5-demethylnobiletin were converted from NOB directly, while 3′,4′-DDMN was converted from 4′-DMN and 3′-DMN (Figure 5). The MRM LC-MS/MS spectrum indicated that a small amount of 5,4′-didemethylnobiletin (8.8 min) and an unknown demethylated NOB (5.3 min) were present.

**FIGURE 6:**
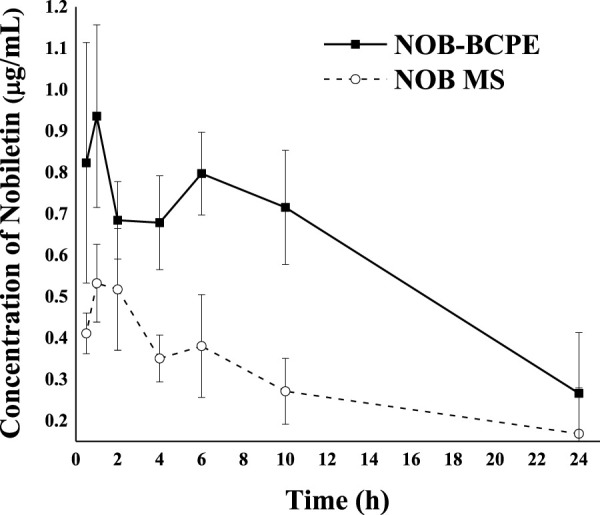
Profiles of plasma concentration of NOB as a function of time after oral administration of 100 mg/kg in the form of BCPE (solid line) and MS (dashed line). Data from five rats are presented as mean ± standard deviation.

The concentration kinetics of NOB and its major metabolite, 4′-DMN, in rat plasma after oral administration of NOB were monitored for 24 h (Figure 6). The pharmacokinetic parameters are summarized in Table 1. The highest peak appeared at a similar time in the NOB-loaded MCT suspension (1.216 ± 0.422 h) and the BCPE (1.152 ± 0.378 h), suggesting that the rates of absorption, metabolism, and excretion were about the same at T_max_. However, the plasma C_max_ of NOB-loaded BCPE (0.936 ± 0.220 μg/mL) was much higher than that of the NOB-loaded MCT suspension (0.532 ± 0.094 μg/mL) at this time. Combined with the *in vitro* results, these findings suggest that BCPE might enhance bioavailability by improving aqueous solubility and controlling release. The plasma concentration of NOB delivered by the MCT suspension dropped dramatically at around 4 h, while the plasma concentration of NOB delivered by BCPE had little change. Then, the plasma concentration of the NOB-loaded BCPE reached a second peak at 6 h. In the TIM-1 GI study of the PMF-loaded whey protein isolate/pectin complexes-stabilized Pickering emulsion, the concentration of NOB in the emulsion reached a peak after 3 h of digestion and then decreased (Wijaya et al., 2020). This phenomenon revealed that BCPE had a sustained release effect. The AUC_0–24_ of NOB in the MCT suspension (6.84 ± 1.48 h*μg/mL) and BCPE (14.19 ± 1.67 h*μg/mL) were significantly different. The relative bioavailability of NOB loaded in BCPE was 2.07 times higher than that suspended in MCT over 24 h. Plasma concentrations of NOB in both BCPE and MCT suspensions returned to similar levels at 24 h.

**TABLE 1:** Pharmacokinetic parameters of NOB in rats after oral administration.

Formulation	NOB dose (mg/kg)	T_max_ (hr)	C_max_ (μg/mL)	AUC_0–24_ (hr × μg/mL)	Relative bioavailability
MCT suspension	100	1.216 ± 0.422	0.532 ± 0.094	6.84 ± 1.48	2.07
Emulsion	100	1.152 ± 0.378	0.936 ± 0.220	14.19 ± 1.67	

In the plasma concentration–time curve, the plasma concentration data suggest a two-compartment model. The extended release of NOB delivered by BCPE could be associated with the viscoelasticity and viscosity of BCPE (Ting et al., 2015). In addition, the composition of the emulsion stabilizer might contribute to different absorption and metabolism rates of NOB (Dickinson, 2009).

The clearance of 4′-DMN in the plasma might be due to 1) further biotransformation to other metabolites, 2) delivery into targeted organs, and 3) excretion in urine. Compared with the *in vivo* biotransformation results of NOB in conventional emulsion, the different pattern of 4′-DMN in rat plasma observed in this study suggested that the emulsion types could be an important factor for the biotransformation *in vivo* (Zhang et al., 2020). Due to the properties of the Pickering emulsion, the particles or complexes were difficult to detach from the interface of water and oil. Therefore, the Pickering emulsion was considered to have a higher stability than conventional emulsions. The complexes consisted of CMI, an anionic derivative of inulin. Given that CMI could be digested by gut microbiota, the upward trend of plasma 4′-DMN within 10–24 h might result from microbial biotransformation and reabsorption from the colon. Future encapsulation strategies might utilize prebiotics as the building blocks of delivery systems to benefit the gut microbiota.

**FIGURE 7:**
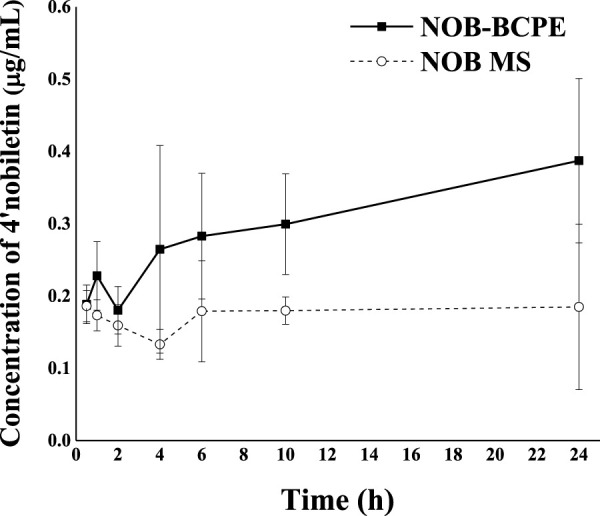
Profiles of plasma concentration of 4′-DMN as a function of time after oral administration of 100 mg/kg in the form of BCPE (solid line) and MS (dashed line), respectively. Data from five rats are presented as mean ± standard deviation.

Figure 7 shows the plasma concentration of 4′-DMN after oral administration of NOB in BCPE and unformulated MCT over 24 h. A much higher level of 4′-DMN was observed in rats receiving BCPE than in rats receiving the MCT suspension, suggesting a higher metabolic rate for NOB-loaded in BCPE than for the MCT suspension. In particular, the concentration of 4′-DMN exhibited an upward trend from 10 h to 24 h. This trend was different from the result of *in vivo* experiments of untreated NOB; the concentration of 4′-DMN sharply decreased from 10 h to 24 h, and 4′-DMN could not be detected in any organ after 24 h, suggesting that BCPE could prolong the residence time of 4′-DMN (Murakami et al., 2002). Such an upward trend of 4′-DMN was not observed in rats receiving the NOB-loaded conventional emulsion (Ting et al., 2015; Zhang et al., 2020). This pattern was probably attributed to the biotransformation of NOB in serum at 6 h, which was the second peak of digestion (Figure 6). Therefore, BCPE could alter the host’s biotransformation of NOB.

## 4 Conclusion

NOB-loaded BCPE was fabricated, and its swallowing and digestive characteristics were evaluated in this study. The characteristics of NOB-loaded BCPE were observed as follows: 1) based on the IDDSI framework, NOB-loaded BCPE was semi-solid and could be swallowed without being chewed. This property could reduce the contact time between NOB and the taste buds to lower the impact of bitterness. In addition, NOB was distributed inside the emulsion, and the ingredients in the water phase might distract from any off-flavor or trigeminal effect. 2) The *in vitro* digestion model revealed that BCPE could enhance the bioaccessibility of NOB by accelerating lipid digestion and micelle formation. 3) The *in vivo* animal study revealed that the plasma concentration of NOB in rats was enhanced after the oral administration of NOB-loaded BCPE. CMI could not be degraded by pepsin, CMI protected BSA from proteolysis, and sustained release of NOB-loaded in BCPE was achieved. 4) The biotransformation content of the major metabolite (4′-DMN) in rat plasma was promoted. BCPE might enhance the bioavailability and promote the biotransformation content of major metabolites by adjusting the micelle formation and intestinal permeability.

The swallowing and digestive profile of BCPE was characterized by the IDDSI framework model, an *in vitro* digestion model, and an *in vivo* animal model, which is useful for precisely applying the system to natural products. According to the results, BCPE might be a potential functional food matrix that can be shaped as needed. It might be a good delivery system for hydrophobic and bitter natural products.

## Data Availability

The original contributions presented in the study are included in the article/Supplementary Material; further inquiries can be directed to the corresponding authors.
